# Thermal and cardiovascular responses to sauna are attenuated by adding cold water immersion to cooling breaks

**DOI:** 10.1113/EP093626

**Published:** 2026-07-02

**Authors:** Tomos F. Owen, Callum J. Giles, Samuel F. Leaney, Geoff B. Coombs, Samuel J. Oliver

**Affiliations:** ^1^ Institute for Applied Human Physiology School of Psychology and Sport Science College of Medicine and Health Bangor University Wales UK

**Keywords:** cardiovascular responses, cooling, core temperature, heat stress, heat therapy, sauna bathing, thermoregulation

## Abstract

The independent effects of different cooling practices, such as cold‐water immersion (CWI), on thermal and cardiovascular responses to sauna remain unclear. This study tested the hypothesis that thermal and cardiovascular responses to sauna are attenuated when CWI was included in a cooling break compared to cooling in air only. In a counterbalanced, crossover design, 16 healthy adults (8 females; age 31 (7) years) participated, on separate visits, in two trials involving 2 × 15 min, 85°C sauna bouts, each followed by a 10‐min cooling break in 23°C air (AIR), or AIR with a 90‐s, 20°C whole‐body CWI. At the end of the second sauna bout, core temperature and heart rate were lower in CWI than AIR (*P* ≤ 0.003, 37.55°C versus 38.03°C, −0.48°C [95% CI: −0.71, −0.25], *d* = 1.28, and 108 bpm versus 125 bpm, −17 bpm [95% CI: −26, −6.6], *d* = 1.05). Skin temperature was also lower in CWI than in AIR (*P* < 0.001). Systolic blood pressure and mean arterial pressure did not differ between conditions (*P ≥* 0.090), whilst diastolic blood pressure was lower in AIR than CWI at the end of cooling breaks and 20 min into recovery (*P* ≤ 0.038). Participants reported feeling less hot and more able to tolerate the second sauna in CWI than in AIR (*P* ≤ 0.002, *d* = 1.27 and 0.97). In conclusion, CWI substantially attenuated thermal and cardiovascular strain and improved the perceptual responses to sauna compared with air cooling alone, making sauna more tolerable and reducing heat illness risks.

## Abstract (highlights)

- **What is the central question of this study?**
  Are thermal and cardiovascular responses to sauna attenuated by the inclusion of a 90‐s, 20°C, cold‐water immersion (CWI) during a cooling break?
- **What is the main finding and its importance?**
  Body temperatures (core and skin), heart rate and subjective ratings of thermal discomfort to sauna are substantially attenuated when CWI is included in sauna cooling breaks, compared to resting for 10 min in air alone. CWI can be incorporated into sauna practice to improve the perceptual experience and reduce heat strain and associated risks of hyperthermia, such as orthostatic intolerance.

## 1 INTRODUCTION

Regular sauna bathing is associated with multiple physical and mental health benefits, including a reduced risk of mental health disorders, cardiovascular and all‐cause mortality (Laukkanen et al., 2015, 2018b), as well as improved physical capacity (Kunutsor et al., 2024) and wellbeing (Strandberg et al., 2018). Sauna bathing typically involves repeated bouts of heat exposure interspersed with cooling breaks, during which individuals adopt different practices such as sitting in ambient air, taking a shower or immersing themselves in cold water (Kukkonen‐Harjula & Kauppinen, 2006; Laukkanen et al., 2018a). Although evidence is emerging that sauna frequency and session duration are associated with health benefits (Laukkanen et al., 2015), the cooling practices in these studies were not reported in detail. Further, research systematically evaluating the influence of different cooling practices during sauna is limited, despite the widespread use and increasing popularity of sauna (Heinonen & Laukkanen, 2018; Laukkanen et al., 2018a).

The underlying mechanisms for the purported health benefits of sauna are multifaceted, but the profound thermoregulatory and cardiovascular challenge is likely a key stimulus for many of the beneficial adaptations (Heinonen & Laukkanen, 2018). Similarly, hyperthermia poses physiological challenges and risks, including orthostatic hypotension, and caution is warranted to prevent excessive heat strain – particularly in individuals with cardiovascular risk factors or disease (Laukkanen et al., 2018a). Cold‐water immersion (CWI) may substantially attenuate thermal and cardiovascular strain during heat exposure due to its greater thermal conductivity compared to air (Tipton et al., 2017). Yet the physiological and perceptual responses to alternating hot and cold exposures during sauna bathing have received limited investigation (Heinonen & Laukkanen, 2018). Kauppinen (1989a, 1989b, 1989c) examined sauna cooling practices; however, unequal heating and cooling durations between conditions limit the ability to isolate the specific effects of CWI relative to other cooling modalities, including ambient air (Kauppinen, 1989a, 1989b, 1989c). As a result, the independent effects of different cooling modalities on thermal and cardiovascular responses to sauna remain unclear. Controlling both sauna and cooling break duration is necessary to accurately determine these effects and to inform safe and effective sauna practices.

Therefore, this study aimed to compare the thermal and cardiovascular responses to sauna when two 15‐min sauna bouts are separated by 10‐min cooling breaks in ambient air or ambient air with a brief CWI. We hypothesised that thermal and cardiovascular responses to sauna would be attenuated when CWI was included in a seated cooling break in air compared to a seated cooling break in air only.

## 2 METHODS

### 2.1 Ethics and informed consent

This project was approved by the Bangor University Academic Research Ethics Committee (no. 2025‐0606). This study was conducted in accordance with the *Declaration of Helsinki* except for prospective registration in a publicly accessible database. Before participation, all individuals provided written informed consent.

### 2.2 Participants

Sixteen healthy adults (8 females, 8 males; age 31 (7) years; height 171 (9) cm; body mass 70 (12) kg; body mass index 24 (3) kg m^−^^2^; physical activity 3622 (3096) MET min week^−^^1^) were recruited. An a priori sample size estimation (G*Power 3.1.9.4) determined that a minimum of 10 participants were required to detect a between‐trial difference in core temperature (*T*_core_) of 0.15°C (Costello et al., 2012), α = 0.05, power = 0.8 and *ƒ* = 0.25). Sixteen participants were recruited as more conservative effects were expected in the present study, due to a shorter immersion and the possibility of warmer lake water temperatures. All participants were healthy and free from known cardiorespiratory, neurological or metabolic conditions, and were not engaged in any form of regular (more than once per week) heat or cold exposure, including sauna and hot bath use.

### 2.3 Study design

**FIGURE 1:**
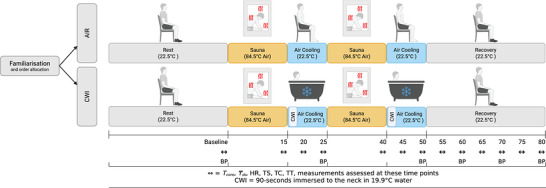
Experimental protocol. AIR, sauna with air cooling breaks trial; BP, blood pressure; CWI, sauna with air and cold‐water immersion cooling breaks trial; HR, heart rate; TC, thermal comfort; *T*_core_, core temperature; TS, thermal sensation; ${\bar{T}}_{\mathrm{sk}}$, mean skin temperature; TT, thermal tolerance. Created with BioRender.com.

Participants completed a familiarisation and then, in a counterbalanced, crossover design, two experimental trials separated by at least 48 h: sauna with air‐cooling (AIR) and sauna with air cooling and a 90‐s CWI. Both trials were conducted at the same time of day (Figure 1) at a Finnish‐style sauna in North Wales (Sawna Bach, Llanberis, UK). Trial order was counterbalanced within each sex. Female participants completed trials 48 h apart, but testing was not standardised to a particular mfenstrual cycle phase. All measurements outside the sauna were conducted in shaded conditions within a portable gazebo positioned near the sauna. Except for the cooling break modality, procedures were identical between trials.

### 2.4 Familiarisation

During familiarisation, participants completed a standardised medical questionnaire, provided written informed consent, and completed the Physical Activity Questionnaire (Department of Health, 2009). Resting blood pressure (BP) was measured in accordance with the European Society of Hypertension practice guidelines (Stergiou et al., 2021) using a validated brachial oscillometric BP monitor (UM‐211, A & D Medical, Abingdon, UK) (Fania et al., 2017). Height and body mass were measured by a wall‐mounted stadiometer (26SM, Sknol, Mumbai, India) and calibrated electronic scales (Seca 635, Hamburg, Germany).

### 2.5 Experimental interventions

Each trial consisted of two 15‐min seated sauna exposures at 84.5 (4.7)°C, followed by 10 min of cooling (Figure 1). In the AIR trial, participants exited the sauna and remained seated in shaded ambient outdoor conditions. The CWI trial was the same as the AIR trial except that after leaving the sauna participants immediately immersed themselves to the neck in 19.9 (3.2)°C water for 90 s and then returned to the shaded seating area for the remainder of the cooling break. In both trials, the sauna and cooling breaks were followed by a 30‐min seated outdoor recovery.

### 2.6 Experimental procedures

Participants were instructed to avoid heat exposure for at least 36 h, strenuous exercise for 24 h, caffeine and alcohol for 12 h, and to eat no more than a light snack for 6 h before each visit, and they confirmed this on a pre‐trial screening form.

Upon arrival, participants changed into swimwear and provided a small (<25 mL) urine sample to assess hydration status using a handheld refractometer (MASTER Refractometer, ATAGO Co., Ltd, Tokyo, Japan). Participants then self‐inserted a rectal thermistor 15 cm beyond the anal sphincter (400‐S, Henleys Medical Supplies Ltd., Welwyn Garden City, UK), and were instrumented with a heart rate (HR) monitor (Polar H10, Polar Electro Oy, Kempele, Finland), and four wireless temperature loggers placed on the skin of the chest, upper arm, thigh and calf (DS1922T‐F5, Maxim Integrated, Wilmington, MA, USA). After 30 min of seated rest, baseline *T*_core_, mean skin temperature (${\bar{T}}_{\mathrm{sk}}$), HR, perceptual responses (thermal sensation, tolerance and comfort; International Organization for Standardization, 2019), and BP were recorded. Body mass was measured in swimwear immediately before entering the sauna.

During the experimental protocol, *T*_core_, HR, perceptual responses and ${\bar{T}}_{\mathrm{sk}}$ were measured at baseline, at the end of each sauna exposure, 5 and 10 min into each cooling break, and every 5 min throughout the 30‐min recovery period. BP was measured at baseline, at the end of each cooling break, and every 10 min during recovery. For the first measurement of each trial, the mean of the second and third readings was used to minimise the alerting response; subsequent measurements were calculated from the mean of three readings. Mean arterial pressure (MAP) was calculated using the equation:

$$\mathrm{MAP}=\left(1/3\times \mathrm{SBP}\right)+\left(2/3\times \mathrm{DBP}\right)$$

where SBP is systolic blood pressure and DBP is diastolic blood pressure. Skin temperatures (*T*_chest_, *T*_arm_, *T*_thigh_ and *T*_calf_) were recorded continuously at 0.1 Hz and then exported to create 1‐min averages. ${\bar{T}}_{\mathrm{sk}}$ was calculated using the four‐site equation (Ramanathan, 1964):

$${\bar{T}}_{\mathrm{sk}}=0.3\left(T_{\mathrm{chest}}+T_{\mathrm{arm}}\right)+0.2\left(T_{\mathrm{thigh}}+T_{\mathrm{calf}}\right)$$

Mean body temperature (${\bar{T}}_{b}$) was calculated using the following equation (Burton, 1935; Lenhardt & Sessler, 2006):

$${\bar{T}}_{b}=\left(0.64\times T_{\mathrm{core}}\right)+\left(0.36\times {\bar{T}}_{\mathrm{sk}}\right)$$

Sauna temperature and humidity were measured at 15 and 40 min using a Ruuvi thermometer (Ruuvi Innovations Ltd, Turku, Finland). Outdoor temperature and humidity were recorded during all outdoor time points, and barometric pressure was measured once at the start of each trial, also using a Ruuvi thermometer. Water temperature was measured during each immersion by a thermistor.

Participants were allowed to drink water ad libitum. After the second cooling break, during the seated recovery phase, participants were permitted to use additional coverings to maintain thermal comfort, reflecting typical real‐world sauna behaviour. Body mass was measured again at the end of recovery, and sweat losses were estimated from the changes in body mass adjusted for fluid intake.

### 2.7 Statistical analysis

All statistical analyses were performed in GraphPad Prism version 10 (GraphPad Software, Boston, MA, USA). A mixed‐effects model with Greenhouse–Geisser correction was used to examine the effects of trial, time and interaction on physiological and perceptual measures, with trial and time included as repeated measures effects. Tukey‐corrected *post hoc* comparisons were applied where an interaction effect was observed. Statistical significance was set at *P *< 0.05, and data in text, figures and table are presented as means (SD), except for the absolute *T*_core_ and HR in the text, which are least square means from the mixed effects model. The size of the between‐trial differences was calculated using Cohen's *d* effect size, with values 0.2, 0.5 and 0.8 interpreted as small, medium and large effects, respectively (Cohen, 1988). Sweat losses and environmental variables were compared between trials using Student's two‐tailed, paired *t*‐test.

## 3 RESULTS

Ambient temperature and humidity (22.5 (5.9)°C, 59.1 (13.5)%) and sauna temperature and humidity (84.5 (4.7)°C, 16.3 (2.5)%) did not differ between trials (*P* = 0.628, 0.158, 0.167 and 0.074, respectively). Due to a technical error, HR data were missing for one female, and ${\bar{T}}_{\mathrm{sk}}$ data were missing for one female and one male during the AIR trial. Pre‐trial hydration status, as indicated by urine specific gravity, was not different between AIR and CWI (AIR 1.008 (0.006), CWI 1.009 (0.006) g mL^−1^, *P *= 0.321).

### 3.1 Thermal and cardiovascular responses to sauna

**FIGURE 2:**
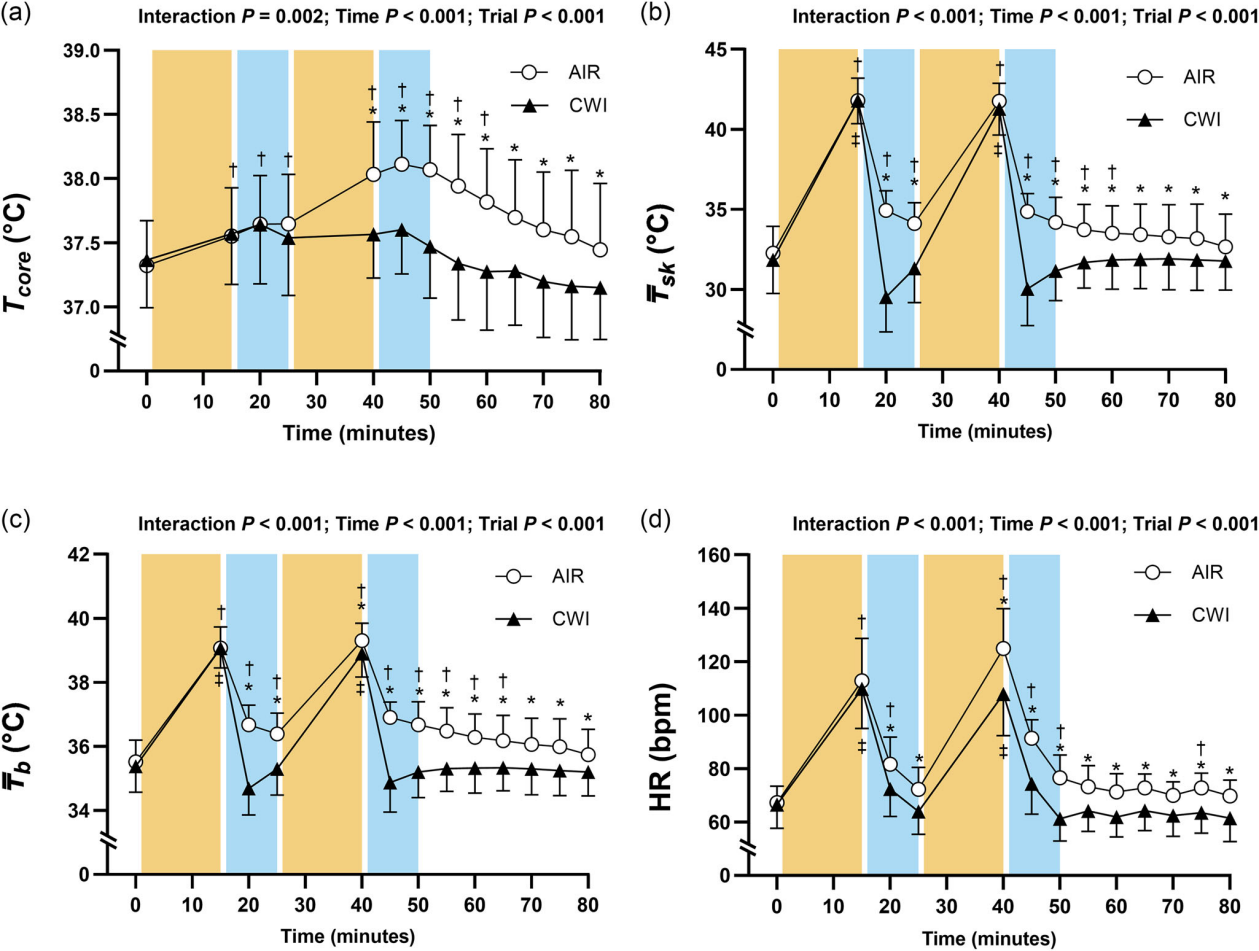
Body core temperature (*T*_core_, a), mean skin temperature (${\bar{T}}_{\mathrm{sk}}$, b), mean body temperature (${\bar{T}}_{b}$, c), and heart rate (HR, d) for sauna with air cooling breaks (AIR; open circles) and sauna with air and cold‐water immersion cooling breaks (CWI; filled triangles). Orange shading denotes sauna exposure; blue shading denotes cooling break. Values are means (SD). *Between‐trial difference (*P *< 0.05); †change from baseline within AIR (*P *< 0.05); ‡change from baseline within CWI (*P *< 0.05).

**FIGURE 3:**
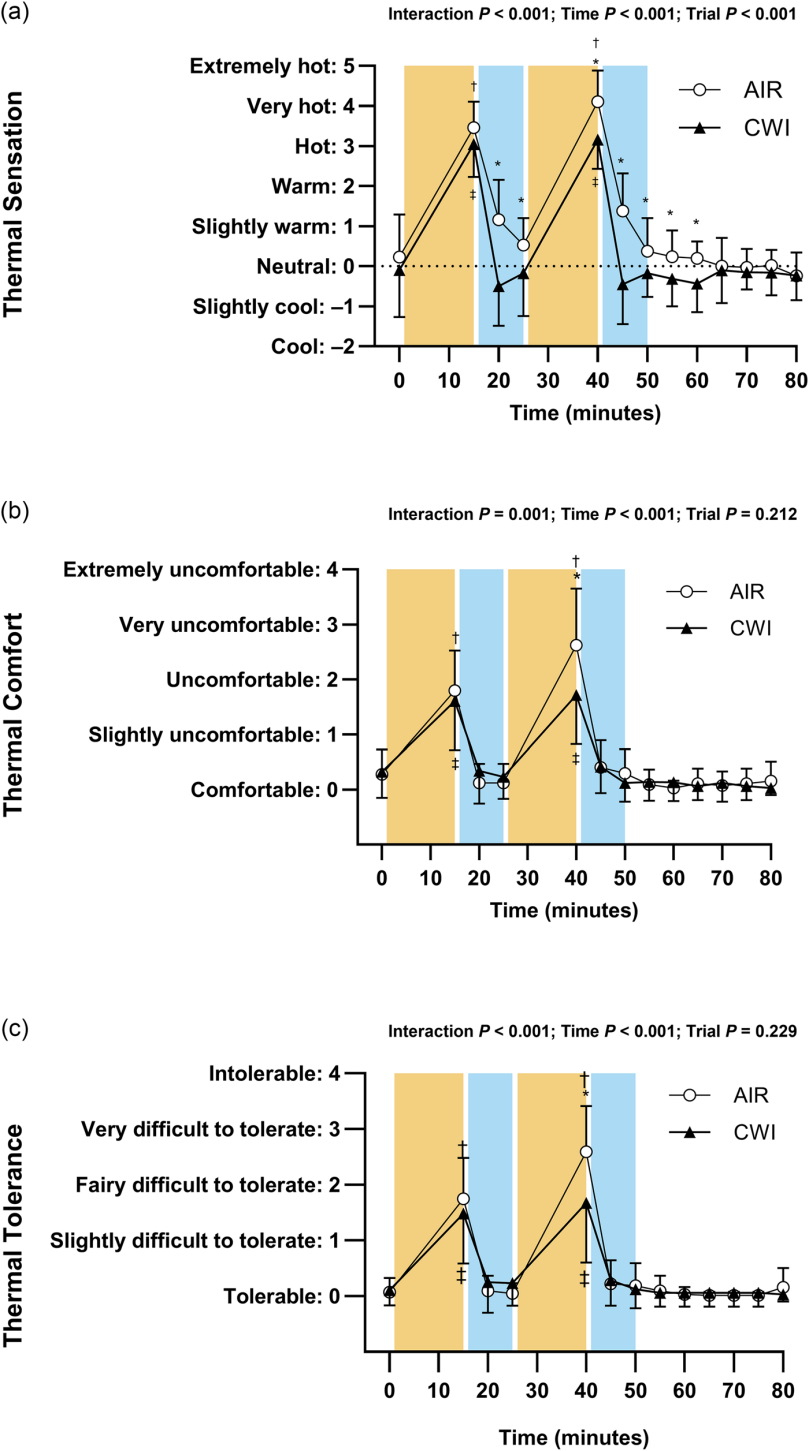
Thermal sensation (a), thermal comfort (b), and thermal tolerance (c) to sauna with air cooling breaks (AIR; open circles) and sauna with air and cold‐water immersion cooling breaks (CWI; filled triangles). Orange shading denotes sauna exposure; blue shading denotes cooling break. Values are mean (SD). *Between‐trial difference (*P *< 0.05); †change from baseline within AIR (*P *< 0.05); ‡change from baseline within CWI (*P *< 0.05).

At baseline, thermal, cardiovascular and perceptual variables were not different between trials (all *P* ≥ 0.372, Table 1; Figures 2 and 3).

**TABLE 1:** Blood pressure responses to sauna with air cooling breaks (AIR) and sauna with air and cold‐water immersion cooling breaks (CWI).

		0 min	25 min	50 min	60 min	70 min	80 min	*P*		
Variable	Trial	Baseline	End of cooling break 1	End of cooling break 2	Recovery +10 min	Recovery +20 min	Recovery +30 min	Time x trial	Time	Trial
SBP (mmHg)	AIR	111 (11)	110 (10)	113 (12)	114 (12)	112 (12)	112 (13)	0.293	0.625	0.990
	CWI	113 (11)	112 (13)	113 (13)	111 (11)	111 (12)	111 (10)			
DBP (mmHg)	AIR	71 (5)	66 (6)†, *	65 (6)*	72 (8)	72 (6)*	74 (6)	<0.001	<0.001	0.034
	CWI	71 (7)	71 (7)	73 (8)	74 (6)	75 (6)	74 (6)			
MAP (mmHg)	AIR	84 (7)	79 (9)	81 (8)	86 (9)	85 (7)	87 (8)	0.090	<0.001	0.245
	CWI	85 (8)	83 (11)	87 (9)	86 (7)	87 (8)	85 (10)			

Data are means (SD). DBP, diastolic blood pressure; MAP mean arterial pressure; SBP, systolic blood pressure.

* between‐trial difference (*P* < 0.05).

^†^ change from baseline within AIR (*P* < 0.05).

Compared with AIR, CWI attenuated the increase in *T*_core_ (Figure 2a), ${\bar{T}}_{\mathrm{sk}}$ (Figure 2b), ${\bar{T}}_{b}$ (Figure 2c) and HR (Figure 2d) to sauna (condition × time interaction, all *P ≤* 0.002, main effect of time, all *P *< 0.001). *T*_core_ increased from baseline to the end of the second sauna in AIR (0.73°C [95% CI: 0.43, 1.03], *d* = 1.92). In contrast, *T*_core_ was not statistically different in CWI from baseline to the end of the second sauna (mean difference, 0.20°C [95% CI: −0.15, 0.55], *d* = 0.56). Subsequently, *T*_core_ was lower in CWI than in AIR from the end of the second sauna (37.55°C vs. 38.03°C, mean difference −0.48°C [95% CI: −0.71, −0.25], *d* = 1.28) to the end of the recovery (all *P* ≤ 0.039), with a maximum mean difference of −0.63°C [95% CI: −0.86, −0.40], *d* = 1.49) at 15 min into the recovery. The rise in ${\bar{T}}_{\mathrm{sk}}$ was similar between trials at the end of each sauna (both *P ≥* 0.151); however, ${\bar{T}}_{\mathrm{sk}}$ was lower in CWI than in AIR during both cooling breaks, and for the duration of the recovery (all *P* ≤ 0.044). After the first cooling break, ${\bar{T}}_{b}$ was lower in CWI than in AIR at all time points from 20 to 80 min (all *P* ≤ 0.019).

HR increased from baseline to the end of the second sauna in AIR (mean difference 57 bpm [95% CI: 43, 71], *d* = 5.06). In comparison, the HR rise in CWI was attenuated from baseline to the end of the second sauna (mean difference, 41 bpm [95% CI: 28, 54], *d* = 3.27). Subsequently, HR was lower in CWI than in AIR during the first cooling break, at the end of the second sauna (108 bpm vs. 125 bpm, mean difference 17 bpm [95% CI: 6.6, 26], *d* = 1.05), and until the end of the recovery (all *P* ≤ 0.007), with a maximum mean difference of 18 bpm (95% CI: 13, 23; *d* = 1.90) at 5 min into the recovery.

SBP and MAP (Table 1) were not different between trials (condition × time interaction, *P ≥* 0.090). In contrast, DBP was reduced from baseline to the end of the first cooling break in AIR (*P* = 0.020; Table 1), and this reduction was attenuated in CWI, as indicated by the lower DBP in AIR than in CWI (condition × time interaction, *P *< 0.001) during the cooling breaks and 20 min into recovery (all *P* ≤ 0.038). Additionally, DBP was not different from baseline at any CWI trial time point (all *P ≥* 0.145). Sweat losses were not different between trials (*P *= 0.271), with AIR averaging 0.72 kg (95% CI: 0.46, 0.98) and CWI averaging 0.56 kg (95% CI: 0.28, 0.84; *n *= 11).

Sauna increased thermal sensation (participants felt warmer, Figure 3a) and decreased thermal comfort (Figure 3b) and thermal tolerance (Figure 3c) (main effect of time, all *P *< 0.001). However, participants reported cooler thermal sensation in CWI than in AIR (condition × time interaction, *P *< 0.001) from 20 to 60 min (all *P ≤* 0.038; Figure 3a), a period that included the second sauna and recovery. Thermal comfort and tolerance were higher in CWI than in AIR at the end of the second sauna (both *P* ≤ 0.001; Figure 3b,c). At the end of the second sauna, thermal sensation, comfort and tolerance were nearly one whole categorical descriptor different in CWI than in AIR (mean differences 0.95 [95% CI: 0.40, 1.50], 0.91 [95% CI: 0.48, 1.30] and 0.92 [95% CI: 0.43, 1.40], respectively; *d* = 1.27, 0.95 and 0.97; all *P* ≤ 0.002).

## 4 DISCUSSION

Typical sauna practice involves intermittent heating interspersed with cooling breaks using a range of modalities, including ambient air exposure and CWI. This study compared the thermal and cardiovascular responses to sauna with a cooling break in ambient air or ambient air with a brief 90‐s CWI. To our knowledge, this is the first study to match trial sauna heating and cooling break durations, which is necessary to determine the independent effects of different cooling modalities. As hypothesised, the inclusion of a brief CWI during seated cooling breaks attenuated thermal and cardiovascular strain and improved thermal comfort during sauna. In terms of statistical and practical significance, the magnitude of the attenuation was large (Cohen's *d* effect sizes ≥0.9), with *T*_core_ reduced by 0.48°C, heart rate by 17 bpm, and thermal perceptual ratings by about 1 categorical descriptor. Further, whilst the inclusion of CWI attenuated ${\bar{T}}_{\mathrm{sk}}$, ${\bar{T}}_{b}$, HR and BP responses to sauna, the rise in *T*_core_ to sauna was almost completely blunted, highlighting CWI as an effective cooling modality that can be incorporated into sauna practice to improve the perceptual experience, and as a preventative strategy to reduce excessive heat strain, and associated risks of hyperthermia, such as orthostatic intolerance.

The previous single study to examine sauna cooling practices did not match sauna or cooling durations between conditions or statistically compare conditions, limiting the ability to isolate the effect of cooling modality (Kauppinen, 1989a, 1989b, 1989c). Using the same duration cooling breaks and sauna bouts in both trials, the present study demonstrates a clear attenuation of thermal strain – shown by reduced *T*_core_, ${\bar{T}}_{\mathrm{sk}}$ and ${\bar{T}}_{b}$ – in CWI compared to in AIR, which can be attributed to cooling modality (Figure 2). Although not immediately reflected in *T*_core_, the first CWI break was a heat sink that led to a large amount of convective heat loss. Subsequently, the lower ${\bar{T}}_{b}$, mitigated thermal strain during the remainder of the sauna, as shown by reduced *T*_core_ in the second sauna bout, and accelerated post‐sauna recovery (Figure 2). This finding is consistent with evidence showing that CWI enables substantially greater heat transfer to equivalent temperature air cooling (Smith & Hanna, 1975; Tipton et al., 2017). The present study's findings – that CWI attenuates the rise in HR and reduction in DBP to sauna – align with established physiological responses to isolated passive heat and cold stress (Tipton et al., 2017; Brunt & Minson, 2021). As BP was not measured during sauna exposure, the hypotensive responses during heating could not be assessed, and CWI may therefore not have fully attenuated the reduction in DBP observed in AIR. Nonetheless, the attenuated reduction in DBP during the CWI breaks and recovery compared to AIR highlights that CWI may be a preventative strategy to help mitigate orthostatic hypotension in some cases.

The reduced thermal strain in CWI compared to in AIR produced a less challenging perceptual experience. Using the closest descriptors to the group mean ratings, participants transitioned from feeling ‘very hot’, ‘very uncomfortable’ and ‘very difficult to tolerate’ in AIR, to ‘hot’, ‘uncomfortable’ and ‘fairly difficult to tolerate’ in CWI. Thermal perception is influenced by ${\bar{T}}_{\mathrm{sk}}$ and *T*_core_ (Bulcao et al., 2000); however, as ${\bar{T}}_{\mathrm{sk}}$ was similar between conditions at the end of the second sauna, the lower *T*_core_ in CWI likely drove the observed improvement in perceptual experience.

Our findings highlight that the cooling break type is an important consideration in sauna research and practice, and future research is warranted to determine the influence of other cooling practices on acute thermal and cardiovascular responses to sauna.

### 4.1 Methodological considerations

Conducting the present study in an applied setting enhanced ecological validity but reduced environmental control compared with a laboratory setting; nevertheless, environmental variation was minimal, and importantly, not statistically different between trials. Our unpublished observations indicate that sauna temperature varies by seating position, ranging from ∼70.5°C (lower bench, further from the heat source) to ∼85.5°C (higher bench, closer to the heat source). To minimise this variability, participants’ seating positions were recorded and maintained between trials; however, this was not always feasible because the sauna was open to the public and positions were occasionally occupied on subsequent visits. In addition, customers sometimes poured water onto the sauna stones – a customary practice that transiently increases humidity and thermal stress. As humidity was measured only at the end of each sauna bout, these brief elevations may not have been fully captured. The study was also not prospectively registered in a publicly accessible trial registry.

### 4.2 Perspectives

Sauna practice is varied across cultures and countries, for example, heat source type, temperature and humidity (Laukkanen & Kunutsor, 2024). Our experimental model was aligned with Finnish‐style sauna, which typically consists of repeated cycles of dry heat exposure lasting 5–20 min, interspersed with cooling breaks, during which individuals rest in ambient air, shower, swim or immerse in cold water (Kukkonen‐Harjula & Kauppinen, 2006; Laukkanen et al., 2018a). Accordingly, our findings provide evidence that may help inform sauna practice in healthy adults. Specifically, thermal and cardiovascular strain accumulate with successive heating and cooling cycles, and increased thermal and cardiovascular strain, as well as heat illness and injury risk, should be expected when cooling breaks of equal duration involve air cooling only, rather than more potent cooling modalities such as CWI. Therefore, incorporating CWI into cooling breaks, alongside limiting heat exposure, may be particularly advised for sauna‐naïve individuals or those with reduced heat tolerance. Moreover, as Finnish‐style sauna typically incorporates cooling modalities such as CWI, the more modest increases in thermal and cardiovascular strain observed in CWI compared to in AIR may be a sufficient stimulus to achieve the health benefits associated with regular sauna (Laukkanen et al., 2015, 2018a, 2018b).

Sauna research has identified that the health benefits of sauna are dependent on sauna frequency and duration. For example, in a large prospective cohort data from Finnish middle‐aged men, sauna session durations >19 min were independently associated with reduced cardiovascular mortality (Laukkanen et al., 2015). However, the studies underpinning this threshold do not report whether sauna exposure was continuous, whether breaks were taken, or which cooling modalities – if any – were used. Consequently, it remains unclear whether cooling practice choices modify the long‐term associations between sauna bathing and health. Therefore, those completing future sauna research are encouraged to report cooling break practices in detail (i.e., mode, duration and temperatures). Although no adverse events or complex arrhythmias were reported in one study in patients with stable chronic heart failure and coronary artery disease, a brief CWI following sauna did increase SBP and DBP (Radtke et al., 2016). Future research should clarify the safety of hot–cold transitions in clinical populations, who may stand to benefit most from sauna. In addition, future research should confirm whether the responses observed in the present study in relatively sauna‐naïve individuals may differ in habitual sauna users, who are likely to exhibit greater heat tolerance because of thermoregulatory, cardiovascular and perceptual adaptations to regular heat exposure (McIntyre et al., 2022; Jenkins et al., 2025).

### 4.3 Conclusions

This study provides novel evidence that the thermal and cardiovascular responses to sauna depend on cooling break modality. The inclusion of a brief CWI during cooling breaks substantially attenuated thermal and cardiovascular strain and improved thermal comfort during sauna. CWI can be incorporated into sauna practice to improve the perceptual experience and as a preventative strategy to reduce heat strain and associated risks of hyperthermia, such as orthostatic intolerance.

## AUTHOR CONTRIBUTIONS

Conception and design of the work: Callum J. Giles, Geoff B. Coombs, Samuel J. Oliver. Acquisition, analysis or interpretation of data for the work: Tomos F. Owen, Callum J. Giles, Samuel F. Leaney, Geoff B. Coombs, Samuel J. Oliver. Drafting the work or revising it critically for important intellectual content: Tomos F. Owen, Callum J. Giles, Samuel F. Leaney, Geoff B. Coombs, Samuel J. Oliver. All authors have read and approved the final version of the manuscript and agreed to be accountable for all aspects of the work in ensuring that questions related to the accuracy or integrity of any part of the work are appropriately investigated and resolved. All persons designated as authors qualify for authorship, and all those who qualify for authorship are listed.

## CONFLICT OF INTEREST

The authors declare no conflict of interest. The industry partner did not provide a financial contribution, nor did they have any influence on data interpretation or preparation of the manuscript.

## Data Availability

The data that support the findings of this study are openly available in Figshare, available at https://figshare.com/s/90f62a9bb30dc85473f5.
